# Muscle Mass, Muscle Strength, and Health-Related Quality of Life in Kidney Transplant Recipients: Results of the TransplantLines Biobank and Cohort Study

**DOI:** 10.1016/j.ekir.2024.10.002

**Published:** 2024-10-10

**Authors:** Tim J. Knobbe, Gijs M.M. Lenis, Dirk A.J. van der Vossen, Jory Wentink, Daan Kremer, Evelien E. Quint, Antonio W. Gomes-Neto, Robin P.F. Dullaart, Robert A. Pol, Stefan P. Berger, Casper F.M. Franssen, Stephan.J.L. Bakker, Adrian Post, TransplantLines Investigators, Coby Annema, Hans Blokzijl, Frank AJA. Bodewes, Marieke T. de Boer, Kevin Damman, Martin H. de Borst, Arjan Diepstra, Gerard Dijkstra, Caecilia SE. Doorenbos, Michele F. Eisenga, Michiel E. Erasmus, C Tji Gan, Eelko Hak, Bouke G. Hepkema, Henri GD. Leuvenink, Willem S. Lexmond, Vincent E. de Meijer, Hubert GM. Niesters, L. Joost van Pelt, Robert A. Pol, Robert J. Porte, Adelita V. Ranchor, Jan Stephan F Sanders, Marion J. Siebelink, Riemer JHJA. Slart, Daan J. Touw, Charlotte A. te Velde-Keyzer, Marius C. van den Heuvel, Coretta van Leer-Buter, Marco van Londen, Erik AM. Verschuuren, Michel J. Vos, Rinse K. Weersma

**Affiliations:** 1 Division of Nephrology, Department of Internal Medicine, University Medical Center Groningen, University of Groningen, Groningen, The Netherlands; 2 Department of Surgery, University Medical Center Groningen, University of Groningen, Groningen, The Netherlands; 3 Department of endocrinology, University Medical Center Groningen, University of Groningen, Groningen, The Netherlands

**Keywords:** kidney transplantation, muscle weakness, patient-reported outcome measures, physical fitness, sarcopenia

## Abstract

**Introduction:**

Muscles are crucial for daily activities, and kidney transplant recipients (KTRs) often have reduced muscle mass and strength. We aimed to investigate the potential relationship of muscle mass and strength with physical health-related quality of life (HRQoL) in KTRs.

**Methods:**

Data from the TransplantLines Biobank and Cohort Studies were used. Muscle mass was assessed using appendicular skeletal muscle mass index (ASMI) and 24-hour urinary creatinine excretion rate index (CERI). Muscle strength was assessed by handgrip strength index (HGSI). HRQoL was measured using Short Form 36 physical component score (PCS).

**Results:**

We included 751 KTRs (61% male; mean age, 56 ± 13 years, median of 3 years post-transplant). Ordinary least squares regression analyses demonstrated that lower ASMI, CERI, and HGSI were all nonlinearly associated with lower PCS, independent of potential confounders and each other. Below median values, ASMI, CERI, and HGSI were each associated with PCS; whereas above median values, associations were less pronounced. Compared to the 50th percentile, a decrease to the 10th percentile was associated with a change in PCS of −4.8% for ASMI (*P* = 0.011), of −5.1% for CERI (*P* = 0.008), and −13.2% for HGSI (*P* < 0.001), whereas an increase to the 90th percentile was associated with a change in PCS of only +0.7% for ASMI (*P* = 0.54), of +3.6% for CERI (*P* = 0.05), and −0.4% for HGSI (*P* = 0.73).

**Conclusion:**

Low muscle mass and strength are potentially modifiable risk factors for impaired physical HRQoL in KTRs. The nonlinear associations suggest that KTRs with low muscle mass or strength may particularly benefit from (p)rehabilitation interventions to improve HRQoL.

## Graphical abstract

**Figure:**
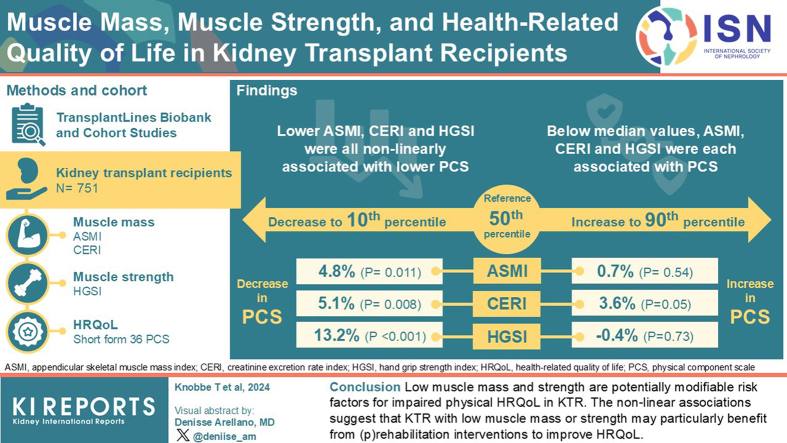

Kidney transplantation is a crucial treatment modality for patients with end-stage kidney disease, offering improved survival rates, cost-effectiveness, and HRQoL compared to dialysis.^1^^,^^2^ Nonetheless, KTRs have lower HRQoL compared to the general population, with notable challenges in terms of physical well-being.^3^ Consequently, there has been a growing recognition among various international working groups that HRQoL serves as a valuable patient-centered outcome for evaluating treatment efficacy and health care quality in chronic kidney disease.^4^, ^5^, ^6^, ^7^, ^8^ This recognition underscores the need to identify novel, modifiable factors that can enhance HRQoL in KTRs.

Muscle weakness represents a potential risk factor that may contribute to lower HRQoL among KTRs, given the critical role of muscle function in almost all daily activities and societal participation, with muscle mass and strength frequently found to be impaired in this population.^9^^,^^10^ Muscle weakness encompasses multiple aspects, including alterations in muscle quantity, muscle quality, and neural activation.^11^ Low muscle mass and strength are independently associated with higher all-cause mortality among KTRs, even after adjusting for each other, highlighting distinct roles in predicting mortality.^10^ However, it remains unknown whether muscle mass and strength are interchangeable or complementary as potential modifiable risk factors for HRQoL. Addressing this knowledge gap is crucial, because it enables the proactive identification of KTRs with suboptimal muscle status and facilitates interdisciplinary interventions to optimize not only longevity but also HRQoL.

The primary aim of the current study, therefore, was to investigate the associations of muscle mass and strength with physical HRQoL in KTRs and specifically their relation to each another. In addition, we assessed whether muscle mass and strength were associated with mental HRQoL. Furthermore, we aimed to place those associations in perspective with the previously found associations of (degree of) airflow limitation and fatigue with HRQoL in KTRs,^12^, ^13^, ^14^ by examining the relationship of muscle mass and strength with airflow limitation and fatigue and exploring whether associations of muscle mass and strength with HRQoL are independent of airflow limitation or fatigue.

## Methods

This study was conducted in accordance with the guidelines for STrengthening the Reporting of OBservational studies in Epidemiology (STROBE, Supplementary Table S1).

### Study Design

Data were extracted from the TransplantLines Biobank and Cohort Study (ClinicalTrials.gov Identifier: NCT03272841) of the University Medical Center Groningen.^15^ Written informed consent was obtained from the KTRs prior to their inclusion. The study was approved by the Medical Ethics Review Board of the University Medical Center Groningen (METc 2014/077) and adheres to the University Medical Center Groningen Biobank Regulation. The clinical and research activities being reported are consistent with the Principles of the Declaration of Istanbul as outlined in the “Declaration of Istanbul on Organ Trafficking and Transplant Tourism” and the World Medical Association Declaration of Helsinki.

For the current cross-sectional study, we included KTRs with a functioning graft ±1 year post transplantation with HRQoL data between June 2015 and November 2022. We excluded patients with missing data on creatinine excretion rate and hand grip strength. In addition, for patients measured on multiple dates, we only incorporated their first measurement. The flow diagram is presented in Supplementary Figure S1.

### Muscle Mass and Strength

Muscle mass was assessed using 2 separate measures. Using a multifrequency bioimpedance analysis device (Quadscan 4000, Bodystat, Douglas, British Isles), we calculated the appendicular skeletal muscle mass following Sergi *et al.*,^16^ which was subsequently divided by height squared to calculate the ASMI, in line with the GLIM and EWSGOP2 criteria.^17^^,^^18^ In addition, we determined muscle mass by measuring the 24-hour creatinine excretion rate from urine collected the day before the visit, directly reflecting the total creatine pool, approximately 95% of which is found in skeletal muscle cells.^19^, ^20^, ^21^, ^22^ Patients received careful instructions for 24-hour urine collection. The excretion rate was divided by height squared to calculate the 24-hour urinary CERI, in line with the use of ASMI.

Muscle strength was evaluated using hand grip strength, which was measured using a hydraulic hand-held dynamometer (Patterson Medical JAMAR 5030J1, Warrenville, Canada).^23^ The measurement was repeated 6 times, alternating between the dominant and nondominant hand with at least 30 seconds between 2 measurements. The highest value was used. Maximum hand grip strength in kg was also divided by height squared to calculate HGSI, in line with the use of ASMI and CERI.

### HRQoL

HRQoL was measured using the Dutch translated Short Form-36, which was sent out shortly before the study visit.^24^^,^^25^ Higher scores indicate better perceived health status. The scores of the various domains result in a physical component score (PCS), which was calculated by averaging the subscales general health, physical health, role limitations due to impairment of physical health and pain, and in a mental component scale (MCS), which was calculated by averaging the subscales emotional well-being, role limitations due to emotional problems, impaired social functioning, and impaired vitality.^13^^,^^24^^,^^25^

### Covariables

Data on age, sex assigned at birth, medical history, and medication use were extracted from patient medical files. Medication use was confirmed during a study visit, when height and weight were measured. Blood samples were collected and laboratory measurements were performed on the same day as the study visit. The estimated glomerular filtration rate (eGFR) was calculated using the 2021 creatinine based Chronic Kidney Disease Epidemiology Collaboration equation.^26^ Protein intake was calculated using 24-hour urinary urea excretion using the Maroni equation.^27^ Diabetes was defined as an HbA1c ≥ 6.5% and/or a nonfasting blood glucose ≥ 200 mg/dl and/or a fasting blood glucose ≥126 mg/dl and/or use of antidiabetic drugs.^28^ The forced expiratory volume in 1 second (FEV_1_) was measured 3 times using a handheld spirometry. The highest value was used. A value below the 5th percentile of an age-, sex-, height- and ethnicity-matched population was applied to indicate airflow limitation, as described in detail previously.^13^^,^^29^ Fatigue was assessed using the Checklist of Individual Strength 20 revised fatigue severity domain.^30^^,^^31^

### Statistical Analyses

Analyses were performed in R version 4.3.0.^32^ In all analyses an alpha of 0.05 was used. Normally distributed data were presented as mean ± SD, nonnormally distributed data as median (interquartile range), and categorical data as number (valid percentage). To determine the best statistical method to analyze the relationship of ASMI, CERI, and HGSI with HRQoL, we first visualized the associations using loess curves, which is a smooth curve that best fits the association between 2 variables in the study population.^33^ We visually identified the values below which ASMI, CERI, and HGSI were strongly positively associated with HRQoL, and values above which there was no clear association. This “threshold” was very close to the median values of ASMI, CERI, and HGSI, as presented in Supplementary Figure S2. We therefore performed ordinary least squares regression analyses, using natural splines with a knot at the median ASMI, CERI, or HGSI value (2 degrees of freedom). To assess the fit of the nonlinear models compared to the linear models, we conducted a likelihood ratio test. The nonlinear models demonstrated a better fit than the linear models regarding the amount of variability explained by the model (*P* < 0.05) Furthermore, we assessed whether further increments in the number of knots (to 2, 3, or 4), led to a better model fit, by comparing the Bayesian Information Criterion and Akaike Information Criterion, and by assessing which model performed better using a likelihood ratio test. Generally, the models with >1 knot did not perform noticeably better than the model with 1 knot regarding Bayesian Information Criterion and Akaike Information Criterion (Supplementary Table S2). In addition, the models did not statistically significantly improve, except for the increment from 1 to 2 knots for the model of CERI and HGSI with PCS, although the curves were comparable (Supplementary Figure S3). Therefore, we proceeded with the least complex model with 1 knot (2 degrees of freedom), to keep the analyses consistent. To account for high outliers in ASMI, CERI, and HGSI, while avoiding the exclusion of participants from the analyses, we defined the upper boundary knot of ASMI, CERI, and HGSI at the 97.5th percentile. Because no low outliers were observed, a lower knot boundary was not defined. After crude analyses, we adjusted for the predefined potential confounders age, sex, eGFR, 24-hour urinary protein excretion, and waist circumference (model 1); history of dialysis, living donor, and time since transplantation (model 2); alcohol use, smoking status, diabetes, and C-reactive protein, hemoglobin (model 3); and protein intake (model 4).^34^, ^35^, ^36^ To assess the interplay between muscle mass and strength, and their association with HRQoL, we additionally adjusted for HGSI (model 5a), ASMI (model 5b) or CERI (model 5c). Because of the collinearity between ASMI and CERI, we did not adjust for both variables in 1 model but adjusted for them separately. Regression analyses results are expressed in standardized betas, which represent how many SDs the depending variable changes with the increase of 1 SD in the continuous independent variable, or by 1 unit for nominal variables. This method enhances the ability to compare effect sizes across independent variables. Potential effect-modification by age, sex, eGFR, and total urinary protein excretion was explored by including product terms in the model. We used linear and logistic regression analyses to assess the associations of ASMI, CERI, and HGSI with FEV_1_, airflow limitation, and fatigue severity; as well as the associations of FEV_1_, airflow limitation, and fatigue severity with HRQoL, because no nonlinearity was observed. Separate analyses were conducted to assess whether the associations of ASMI, CERI, and HGSI with HRQoL remained independent of additional adjustment for these variables. Pairwise exclusion was applied.

### Sensitivity Analyses

Sensitivity analyses include analyses in which we used unindexed appendicular skeletal muscle mass, creatinine excretion rate and handgrip strength, and analyses without knot boundaries of ASMI, CERI, and HGSI. Furthermore, we performed analyses after excluding KTRs with an eGFR ≤ 30 ml/min per 1.73 m^2^ to validate that our results were not driven by KTRs with the most severely impaired kidney function, which typically have the lowest endogenous creatine synthesis.^37^, ^38^, ^39^ In addition, we performed analyses after excluding KTRs aged ≥75 years, to ensure that the results were not driven by the oldest KTRs within our sample, who physiologically have a lower amount of muscle mass and strength.

## Results

A CONSORT 2010 flow diagram on patient-flow through the study is presented in Supplementary Figure S1. We included 751 KTRs ≥1 year after transplantation. The majority were males (61%), and the mean age was 56 ± 13 years. The median time after transplantation was 3 (1–10) years, 68% had a history of dialysis, mean eGFR was 55 ± 18 ml/min per 1.73 m^2^, and median total urinary protein excretion was 0.17 (0.12–0.28) g/24 h. Mean ASMI was 7.1 ± 1.2 kg/m^2^, mean CERI was 4.1 ± 1.1 mmol/24-h/m^2^, and mean HGSI was 12.4 ± 3.4 kg/m^2^ (Table 1). ASMI, CERI, and HGSI were all significantly higher among males compared to females (Supplementary Table S3). The distributions of ASMI, CERI, and HGSI among males and females are presented in Supplementary Figure S4. KTRs included in the analyses were generally comparable to those excluded from the analyses (Supplementary Table S4).

**Table 1:** Characteristics of KTRs

Characteristics	KTRs *N* = 751
Demographics	
Female sex, *n* (%)	289 (39)
Age, yrs	56 ± 13
Weight, kg	82 ± 16
Height, cm	174 ± 9
Waist, cm	99 ± 14
Body surface area, m^2^	2.0 ± 0.2
Body mass index, kg/m^2^	27 ± 5
Diabetes, *n* (%)	210 (28)
Time since transplantation, *n* (%)	
≤2 yrs	321 (43)
2–5 yrs	108 (14)
≥5 yrs	322 (43)
History of dialysis, *n* (%)	509 (68)
Living donor, *n* (%)	416 (55)
Lifestyle factors	
Alcohol use, *n* (%)	
No	286 (38)
<7 units/wk	309 (41)
≥7 units/wk	156 (21)
Smoking status, *n* (%)	
Never smoked	351 (47)
Past smoker	306 (41)
Current smoker	94 (13)
Protein intake, g/d	84 ± 22
Laboratory measurements	
Hemoglobin, mmol/l	8.4 ± 1.1
Leukocyte count, 10^9^/l	7.6 ± 2.2
C-reactive protein, mg/l	1.8 (0.7–4.4)
Creatinine, μmol/l	123 (103–152)
eGFR, ml/min per 1.73 m^2^	55 ± 18
Albumin, g/l	44 ± 4
Total protein excretion, g/24 h	0.17 (0.12–0.28)
Medication use	
Prednisolone, *n* (%)	733 (98)
Calcineurin inhibitor, *n* (%)	618 (82)
Proliferation inhibitor, *n* (%)	643 (86)
mTOR inhibitor, *n* (%)	28 (4)
Muscle mass and strength	
ASM, kg	21.4 ± 4.7
ASMI, kg/m^2^	7.1 ± 1.2
CER, mmol/24 h	12.5 ± 3.8
CERI, mmol/24 h/m^2^	4.1 ± 1.1
HGS, kg	38.1 ± 12.2
HGSI, kg/m^2^	12.4 ± 3.4

Normally distributed data were presented as mean ± SD, nonnormally distributed data as median (interquartile range) and categorical data were presented as number (valid %). No data was missing.

ASM(I), appendicular skeletal muscle mass (index); CER(I), creatinine excretion rate (index); eGFR, estimated glomerular filtration rate; HGS(I), hand grip strength (index); KTR, kidney transplant recipients; mTOR, mammalian target of rapamycin.

### Association of Muscle Mass and Strength with Physical HRQoL

**Figure 1:**
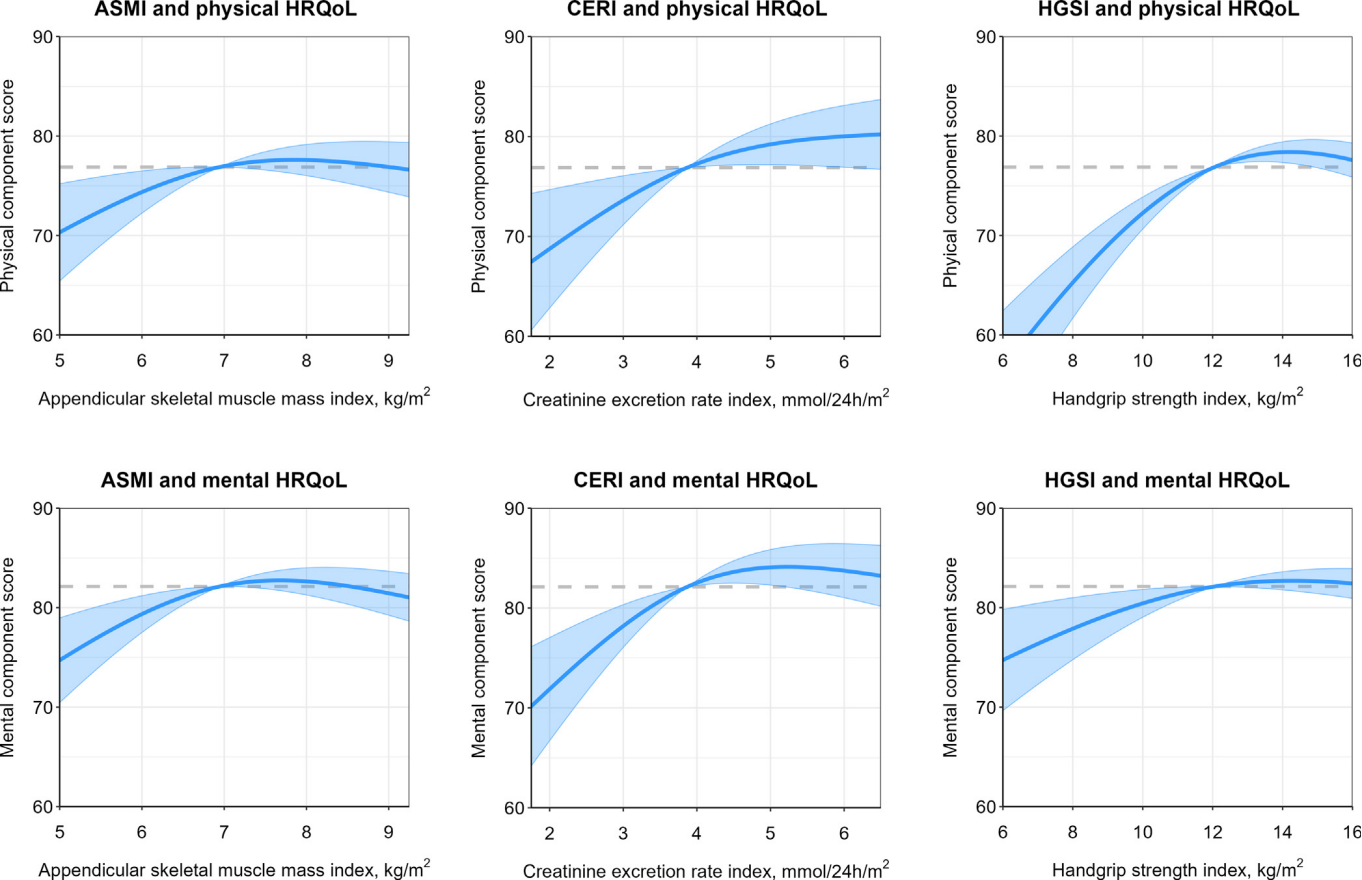
Graphical representation of the associations of ASMI, CERI, and HGSI with physical and mental HRQoL. These graphs represent the fitted splines corresponding to the coefficients of the models reported in Supplementary Table S6, providing a visual representation of the relationships modelled in the regression analysis. The nonlinear nature of the associations was modelled using natural spline terms with 2 degrees of freedom for ASMI, CERI, and HGSI. All analyses were adjusted for age, sex, eGFR, total protein excretion in 24-hours, waist circumference, history of dialysis, living donor, time since transplantation, alcohol use, smoking status, diabetes, C-reactive protein, hemoglobin, protein intake, and CERI or HGSI. Figures illustrating the associations of HGSI adjusted for ASMI are not presented, because these figures are comparable to those adjusted for CERI (Table 2). The shaded area represents the pointwise 95% confidence interval. ASMI, appendicular skeletal muscle mass index; CERI, creatinine excretion rate index; HGSI, hand grip strength index; HRQoL, health-related quality of life.

Mean PCS score was 70 ± 21. We observed nonlinear associations of ASMI, CERI, and HGSI with PCS. Before reaching the median, lower values of ASMI, CERI, and HGSI were all associated with lower PCS. However, beyond the median, these associations were less pronounced. There was no effect modification according to age, sex, eGFR, and urinary protein excretion (Supplementary Table S5). After extensive adjustments for potential confounders, these associations remained virtually unchanged (model 1–4). To explore the potential interplay between muscle mass and strength in relation to PCS, we further adjusted for HGSI, ASMI, or CERI (model 5a–c), and observed that all parameters remained associated with PCS before the median; however, that HGSI appeared more strongly associated with PCS than ASMI and CERI, as presented in Supplementary Table S6 and illustrated in Figure 1 (top panels). Based on the adjusted models, a decrease from the median to the 10th percentile was associated with a PCS change of −4.8% for ASMI (*P* = 0.011), −5.1% for CERI (*P* = 0.008) and −13.2% for HGSI (*P* < 0.001), whereas an increase from the median to the 90th percentile was associated with a change of +0.7% for ASMI (*P* = 0.22), +3.6% for CERI (*P* = 0.05) and −0.4% for HGSI (*P* = 0.73), as presented in Table 2.

**Table 2:** Predicted changes in physical and mental HRQOL based on changing from median to 10th or 90th percentile of ASMI, CERI or HGSI, respectively

Model 4 +	ASMI Adjustment for HGSI			CERI Adjustment for HGSI			HGSI					
							Adjustment for ASMI			Adjustment for CERI		
	Change	% Change	*P*-value	Change	% Change	*P*-value	Change	% Change	P-value	Change	% change	*P*-value
Physical component score												
10th percentile	−3.7	−4.8%	0.011	−3.9	−5.1%	0.008	−10.2	−13.2%	<0.001	−10.2	−13.2%	<0.001
Median	Ref	Ref	Ref	Ref	Ref	Ref	Ref	Ref	Ref	Ref	Ref	Ref
90th percentile	+0.5	+0.7%	0.54	+2.8	+3.6%	0.05	−0.1	−0.1%	0.94	−0.3	−0.4%	0.73
Mental component score												
10th percentile	−4.1	−5.0%	0.005	−4.7	−5.7%	<0.001	−3.8	−4.6%	0.010	−3.7	−4.5%	0.010
Median	Ref	Ref	Ref	Ref	Ref	Ref	Ref	Ref	Ref	Ref	Ref	Ref
90th percentile	+0.2	+0.2%	0.83	+1.7	+2.2%	0.15	+0.7	+0.1%	0.99	−0.1	−0.1%	0.95

The change column refers to the absolute change when going from median to 10th or 90th percentile of ASMI, CERI or HGSI, respectively. The relative change column is defined as the absolute change divided by the value at the median times 100%. The *P*-values were calculated using t-statistics and pooled standard errors. Variables included in model 4 are age, sex, estimated glomerular filtration rate, total protein excretion in 24-hours, waist circumference, history of dialysis, living donor, time since transplantation, alcohol use, smoking status, diabetes, C-reactive protein, hemoglobin and protein intake. Because of collinearity between ASMI and CERI, both variables were not adjusted in one model, but were adjusted separately.

ASMI, appendicular skeletal muscle mass index; CERI, creatinine excretion rate index; HGSI, hand grip strength index; HRQoL, health-related quality of life; Ref, Reference.

### Association of Muscle Mass and Strength With Mental HRQoL

Mean MCS score was 77 ± 17. Similar to PCS, we observed nonlinear associations of ASMI, CERI, and HGSI with PCS. Below the median, AMSI, CERI, and HGSI were all associated with lower MCS. However, beyond the median, these associations were less pronounced. Again, no effect modification according to age, sex, eGFR and urinary protein excretion was found (Supplementary Table S5). Associations remained virtually unchanged after extensive adjustment for potential confounders. All parameters remained associated with MCS after further adjustment for HGSI, ASMI, or CERI; however, in contrast to PCS, ASMI and CERI appeared more strongly associated with MCS than HGSI, as presented in Supplementary Table S6 and illustrated in Figure 1 (bottom panels). Based on the adjusted models, a decrease from the median ASMI, CERI, and HGSI to the 10th percentile was associated with an MCS change of −5.0% for ASMI (*P* = 0.005), −5.7% for CERI (*P* < 0.001) and −4.5% for HGSI (*P* = 0.010), whereas an increase from the median to the 90th percentile was associated with a change of +0.2% for ASMI (*P* = 0.83), +2.2% for CERI (*P* = 0.15) and −0.1% for HGSI (*P* = 0.95), as presented in Table 2.

### Airflow Limitation and Fatigue

Mean FEV_1_ was 2.9 ± 0.8 L; 26% met the criterion of airflow limitation. Higher ASMI and HGSI were associated with higher FEV_1_ (standardized beta = 0.11 [0.04–0.18], *P* = 0.002 and standardized beta = 0.14 [0.07–0.20], P < 0.001, respectively). The associations of ASMI, CERI, and HGSI with PCS remained independent of adjustment for FEV_1_. However, the association of FEV_1_ with PCS weakened and lost statistical significance in these analyses. FEV_1_ was not associated with MCS; and the associations of ASMI, CERI, and HGSI with MCS remained independent of adjustment for FEV_1_ (Supplementary Table S7). Analyses with airflow limitation yielded comparable results, showing consistent associations of ASMI, CERI, and HGSI with PCS and MCS, all independent of airflow limitation (Supplementary Table S8).

Mean fatigue severity score was 27.4 ± 13.1. Only HGSI was significantly associated with fatigue severity (standardized beta = −0.12 [−0.21 to −0.03], *P* = 0.011). After adjusting for fatigue severity, the associations of ASMI, CERI, and HGSI with PCS and MCS all weakened, but remained statistically significant, except for the association between HGSI and MCS, which lost significance. The association of fatigue severity with both MCS and PCS did not change substantially compared to the analyses without ASMI, CERI, or HGSI included in the model (Supplementary Table S9).

### Robustness of Associations with HRQoL

In sensitivity analyses using unindexed variables, or without boundary knots, the associations of ASMI, CERI, and HGSI with both PCS and MCS remained unchanged. In sensitivity analyses after excluding KTRs with an eGFR ≤ 30 ml/min per 1.73 m^2^ and analyses after excluding KTRs aged ≥ 75 years, the associations of HGSI with both PCS and MCS were notably strengthened. The associations of ASMI and CERI were weaker in those subgroup analyses, with the exception for the association of CERI with PCS in analyses after excluding KTRs with an eGFR < 30 ml/min per 1.73 m^2^, which was strengthened. A tabulated overview of the sensitivity analyses is shown in Supplementary Table S10.

## Discussion

This large cross-sectional study shows that lower muscle mass and strength, assessed by ASMI, CERI, and HGSI are strongly nonlinearly associated with lower physical and mental HRQoL in KTRs, independent of potential confounders. Below the median, lower muscle mass and strength were associated with lower physical and mental HRQoL; whereas above the median, the associations between muscle mass and strength with physical and mental HRQoL were far less pronounced. After adjusting for each other, muscle mass and strength remained associated with physical and mental HRQoL. However, muscle strength appeared to be more strongly associated with physical HRQoL, whereas muscle mass appeared to be more strongly associated with mental HRQoL. These associations were independent of (degree of) airflow limitation and fatigue, except the association between muscle strength and mental HRQoL, which lost significance. Notably, fatigue remained significantly associated with both mental and HRQoL in all models, whereas the associations of (the degree of) airflow limitation did not remain significantly associated.

Our overall findings are in line with results of a smaller cohort of KTRs, where muscle mass and strength were associated with physical and mental HRQoL.^35^ The authors concluded that their results were hampered by the limited sample size of 128 KTRs. Similarly, another study that analyzed HRQoL trajectories over the first 3 years posttransplantation of 337 KTRs found that self-reported muscle weakness was the most important covariable to predict the low cluster of physical HRQoL.^40^ Notably, muscle weakness was also associated with mental HRQoL, though to a lesser extent. The current study corroborates their findings with a large KTRs cohort, and expands the knowledge on the topic. Although the authors adjusted for a great variety of potential confounders, the nonlinearity of the associations was not addressed.

**Figure 2:**
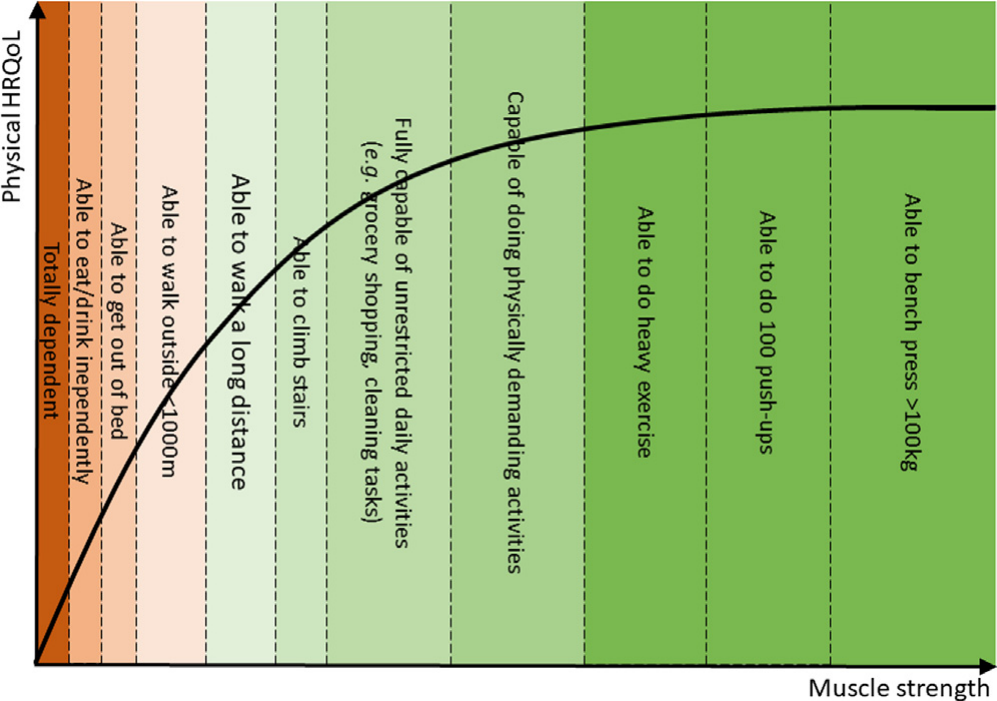
Conceptual schematic illustration of the nonlinear association of muscle strength with physical HRQoL. An increase in muscle strength up to a certain threshold significantly improves physical HRQoL as individuals will be capable of doing activities which are important in daily life. Once this threshold is reached, additional muscle strength does not translate into substantial gains in physical HRQoL, because the activities that are possible with this extra muscle strength are typically not necessary in everyday life. ASMI, appendicular skeletal muscle mass index; CERI, creatinine excretion rate index; HGSI, handgrip strength index; HRQoL, health-related quality of life.

Similar to the association of muscle mass and strength with mortality, we found that lower muscle mass and strength were both associated with lower HRQoL, and that these measurements were complementary to each other.^10^ However, unlike the linear association observed with mortality, the relationship of muscle mass and strength with HRQoL was best modelled using a nonlinear association. This nonlinear association indicates that an increase in muscle mass or strength above the median is barely associated with HRQoL, but that muscle mass and strength values below the median are strongly associated with impaired HRQoL. This may be of importance when selecting patients for interdisciplinary interventions. We observed that females on average have significantly lower muscle strength. Their muscle strength may thus more frequently be at levels that could impair activities of daily living, potentially contributing significantly to a lower physical HRQoL. It is unlikely that sex differences underlie this observation, because no interaction with sex was observed. This indicates that the nonlinear associations with HRQoL are not different between females and males, and these associations remained generally unchanged after adjusting for sex. Our hypothesis is that a certain degree of muscle strength is required for everyday tasks and to perform or participate in (social) activities (e.g., the ability to get out of bed, or to walk outside). If the additional muscle strength enables individuals to perform such activities that were previously impossible, this increase in muscle strength significantly impacts their physical HRQoL. However, once individuals have sufficient muscle strength to handle daily activities, including those that are physically demanding, any surplus in muscle strength beyond this threshold does not significantly influence their everyday life or consequently enhance their physical HRQOL. For example, having the muscle strength to walk outside, enables one to attend social activities and enhance independence. In contrast, having the muscle strength to perform push-ups does not typically contribute to everyday tasks, thus such additional muscle strength does not necessarily result in a gain in physical HRQoL. This concept is schematically illustrated in Figure 2.

Muscle mass demonstrated the strongest association with mental HRQoL, when compared to muscle strength, especially at the lowest values. Creatinine excretion is a direct reflection of the creatine pool, given the fact that 1.7% of the creatine pool is daily converted to creatinine and excreted into urine.^39^^,^^41^ Whereas over 95% of the creatine pool is found in the skeletal muscles, the brain is also highly reliant on creatine, because the brain consumes approximately 20% of total resting energy.^39^^,^^42^ A higher CERI may partially reflect a higher degree of endogenous creatine synthesis and/or creatine intake in the diet, both of which can lead to maintenance of an appropriate creatine concentration in the brain, which may be important for mental HRQoL. The rate of endogenous creatine synthesis depends mostly on the enzyme arginine:glycine amidinotransferase, which is predominantly expressed in the proximal tubular cells of the kidneys.^38^^,^^39^ The importance of endogenous creatine synthesis for the association of CERI with HRQoL is supported by the sensitivity analyses after excluding patients with the lowest kidney function (and thus lowest endogenous creatine synthesis), in which the associations of CERI with HRQoL were weakened.

We previously demonstrated that FEV_1_ was strongly associated with physical HRQoL in KTRs, and that this association was stronger than that of airflow limitation, defined as an FEV_1_ below the 5th percentile of an age-, sex-, height- and ethnicity-matched reference population.^13^ In the current study, we found that muscle mass and strength appear to be key determinants of FEV_1_, and to a lesser extent, of airflow limitation. This is likely because muscles are essential for forceful exhalation, which is required to measure FEV_1_. Notably, we observed that muscle mass and strength were, independently of FEV_1_ or airflow limitation, associated with physical HRQoL. In contrast, neither FEV_1_ nor airflow limitation remained independently associated with physical HRQoL in these statistical models. This suggests that the previously suggested impact of (the degree of) airflow limitation on physical HRQoL may be explained by muscle mass and strength, and that the association of muscle mass and strength with physical HRQoL is noticeably stronger than that of (the degree of) airflow limitation.

Fatigue is considered one of the most important determinants of poor HRQoL in KTRs.^12^^,^^14^ In the current study, we confirmed that fatigue is strongly associated with lower physical and mental HRQoL. We found that this association is independent of muscle mass or strength, because adjusting for these factors did not substantially change the association. In contrast, the associations of muscle mass and strength with HRQoL weakened notably, although most of the associations remained significant. Fatigue appears to be an independent covariate that is associated with HRQoL, independent of muscle mass and strength, and explained some of the variance in HRQoL that was attributable to muscle mass and strength without taking fatigue into account. However, muscle mass and strength still retain independently, albeit weaker, associated with HRQoL, suggesting distinct roles in influencing HRQoL beyond the effects on fatigue.

A strength of the current study is the large, well-characterized cohort of KTRs. Furthermore, the dual assessment of muscle mass using 2 distinct parameters enhances the reliability and validity of our findings, offering a more thorough characterization of muscle mass than a single assessment could achieve. In addition, we conducted thorough and comprehensive analyses, considering multiple statistical methods and ensuring robustness of our results. For instance, we identified the optimal threshold based on a loess curve. This nonparametric technique results in a smooth curve that best fits the association between 2 variables^33^ and enabled us to visually identify threshold values for ASMI, CERI, and HGSI below which these were significantly associated with HRQoL and therefore could be used as target values to aim for in KTRs with low ASMI, CERI, and HGSI. However, there are a few limitations we acknowledge. Due to the observational nature of the study design, no causal relationship between measures of muscle mass or strength with HRQoL can be established. Therefore, we cannot distinguish whether the relationship of muscle mass and strength with HRQoL is causal or associative. Because this study used a preexisting cohort of 751 patients, the sample size was fixed, and formal power calculations were not conducted prior to the analyses. Although the sample size of the cohort is relatively large for the transplant field, it may be insufficient to detect small statistical associations. In addition, the inclusion of multiple predictors adds complexity to the model, which may also contribute to insufficient power, particularly with regard to identifying significant interactions. Trials are needed to determine whether interdisciplinary interventions that improve muscle mass and strength can improve both clinical outcomes and HRQoL in KTRs.

In conclusion, muscle mass and strength may be potentially modifiable risk factors for impaired physical and mental HRQoL in KTRs. Both muscle mass and strength were complementarily associated with HRQoL. These results suggest that muscle status is particularly relevant for HRQoL in KTRs with low muscle mass and strength. This insight is pivotal for identifying patients who could benefit the most from targeted (pre)rehabilitation programs aimed at enhancing their HRQoL.

## Appendix

### List of the TransplantLines Investigators

Coby Annema, Hans Blokzijl, Frank AJA Bodewes, Marieke T de Boer, Kevin Damman, Martin H de Borst, Arjan Diepstra, Gerard Dijkstra, Caecilia SE Doorenbos, Michele F Eisenga, Michiel E Erasmus, C Tji Gan, Eelko Hak, Bouke G Hepkema, Henri GD Leuvenink, Willem S Lexmond, Vincent E de Meijer, Hubert GM Niesters, L Joost van Pelt, Robert A Pol, Robert J Porte, Adelita V Ranchor, Jan-Stephan F Sanders, Marion J Siebelink, Riemer JHJA Slart, Daan J Touw, Charlotte A te Velde-Keyzer, Marius C van den Heuvel, Coretta van Leer-Buter, Marco van Londen, Erik AM Verschuuren, Michel J Vos, and Rinse K Weersma. Authors TJK, DK, AWGN, SPB, and SJLB are TransplantLines Investigators.

## Disclosure

All the authors declared no competing interests.

## Supplementary Material

Supplementary File (PDF) **Characteristics of muscle mass and muscle strength stratified by sex**. **Figure S1.** CONSORT 2010 flow Diagram. **Figure S2.** Loess curves of the associations between ASMI, CERI, or HGSI with physical and mental HRQoL. **Figure S3.** Curves of the model including CERI and HGSI with physical HRQoL with 2 (blue) or 3 (red) degrees of freedom. **Figure S4.** Data distribution of ASMI, CERI, and HGSI stratified by sex. **Table S1.** STROBE Statement - Checklist of items that should be included in reports of cohort studies. **Table S2.** Model fit parameters according to the degrees of freedoms in the nonlinear models. **Table S3.** Characteristics of KTRs excluded from and included in analyses. **Table S4.** Characteristics of KTRs excluded from and included in analyses. **Table S5.** Statistical significance of potential effect modifications. **Table S6.** Ordinary least squares regression analyses on the association of ASMI, CERI, and HGSI with HRQoL. **Table S7.** Associations of ASMI, CERI, or HSGI with FEV_1_, and their relationship with HRQoL in mutually adjusted analyses. **Table S8.** Associations of ASMI, CERI, or HSGI with airflow limitation and their relationship with HRQoL in mutually adjusted analyses. **Table S9.** Associations of ASMI, CERI, or HSGI with fatigue severity and their relationship with HRQoL in mutually adjusted analyses. **Table S10.** Analyses to assess the robustness of ordinary least squares regression analyses on the association of muscle mass and muscle strength with HRQoL.

## Footnotes

Supplementary File (PDF)

**Characteristics of muscle mass and muscle strength stratified by sex.**
**Figure S1.** CONSORT 2010 flow Diagram. **Figure S2.** Loess curves of the associations between ASMI, CERI, or HGSI with physical and mental HRQoL. **Figure S3.** Curves of the model including CERI and HGSI with physical HRQoL with 2 (blue) or 3 (red) degrees of freedom. **Figure S4.** Data distribution of ASMI, CERI, and HGSI stratified by sex. **Table S1.** STROBE Statement - Checklist of items that should be included in reports of cohort studies. **Table S2.** Model fit parameters according to the degrees of freedoms in the nonlinear models. **Table S3.** Characteristics muscle mass and muscle strength stratified by sex. **Table S4.** Characteristics of KTRs excluded from and included in analyses. **Table S5.** Statistical significance of potential effect modifications. **Table S6.** Ordinary least squares regression analyses on the association of ASMI, CERI, and HGSI with HRQoL. **Table S7.** Associations of ASMI, CERI, or HSGI with FEV_1_, and their relationship with HRQoL in mutually adjusted analyses. **Table S8.** Associations of ASMI, CERI, or HSGI with airflow limitation and their relationship with HRQoL in mutually adjusted analyses. **Table S9.** Associations of ASMI, CERI, or HSGI with fatigue severity and their relationship with HRQoL in mutually adjusted analyses. **Table S10.** Analyses to assess the robustness of ordinary least squares regression analyses on the association of muscle mass and muscle strength with HRQoL.
