# Supplementary material for: Muscle Mass, Muscle Strength, and Health-Related Quality of Life in Kidney Transplant Recipients: Results of the TransplantLines Biobank and Cohort Study
Source: Kidney Int Rep. 2024 Oct 10;10(1):99–108. doi: 10.1016/j.ekir.2024.10.002 (PMC11725826; doi:10.1016/j.ekir.2024.10.002)
Supplement: Supplementary File (PDF) — Characteristics of muscle mass and muscle strength stratified by sex. Figure S1. CONSORT 2010 flow Diagram. Figure S2. Loess curves of the associations between ASMI, CERI, or HGSI with physical and mental HRQoL. Figure S3. Curves of the model including CERI and HGSI with physical HRQoL with 2 (blue) or 3 (red) degrees of freedom. Figure S4. Data distribution of ASMI, CERI, and HGSI stratified by sex. Table S1. STROBE Statement - Checklist of items that should be included in reports of cohort studies. Table S2. Model fit parameters according to the degrees of freedoms in the nonlinear models. Table S3. Characteristics of KTRs excluded from and included in analyses. Table S4. Characteristics of KTRs excluded from and included in analyses. Table S5. Statistical significance of potential effect modifications. Table S6. Ordinary least squares regression analyses on the association of ASMI, CERI, and HGSI with HRQoL. Table S7. Associations of ASMI, CERI, or HSGI with FEV1, and their relationship with HRQoL in mutually adjusted analyses. Table S8. Associations of ASMI, CERI, or HSGI with airflow limitation and their relationship with HRQoL in mutually adjusted analyses. Table S9. Associations of ASMI, CERI, or HSGI with fatigue severity and their relationship with HRQoL in mutually adjusted analyses. Table S10. Analyses to assess the robustness of ordinary least squares regression analyses on the association of muscle mass and muscle strength with HRQoL. [file mmc1.pdf]

Supplementary Material

Supplementary Table S1. STROBE Statement—Checklist of items that should be included in reports of cohort studies

	Item No	Recommendation	Page No
**Title and abstract**	1	(*a*) Indicate the study's design with a commonly used term in the title or the abstract	N/A
		(*b*) Provide in the abstract an informative and balanced summary of what was done and what was found	Abstract
**Introduction**			
Background/rationale	2	Explain the scientific background and rationale for the investigation being reported	Intro par 1-3
Objectives	3	State specific objectives, including any prespecified hypotheses	Intro par 3
Methods			
Study design	4	Present key elements of study design early in the paper	Methods 'study design'
Setting	5	Describe the setting, locations, and relevant dates, including periods of recruitment, exposure, follow-up, and data collection	Methods 'study design'
Participants	6	(*a*) Give the eligibility criteria, and the sources and methods of selection of participants. Describe methods of follow-up	Methods 'study design', SF1 Follow-up N/A
		(*b*) For matched studies, give matching criteria and number of exposed and unexposed	N/A
Variables	7	Clearly define all outcomes, exposures, predictors, potential confounders, and effect modifiers. Give diagnostic criteria, if applicable	Methods 'exposures', 'outcomes' and 'covariables', ST4
Data sources/ measurement	8*	For each variable of interest, give sources of data and details of methods of assessment (measurement). Describe comparability of assessment methods if there is more than one group	Methods 'exposures', 'outcomes' and 'covariables'
Bias	9	Describe any efforts to address potential sources of bias	Methods 'statistical analyses', 'sensitivity analyses'
Study size	10	Explain how the study size was arrived at	Methods 'study design', SF1
Quantitative variables	11	Explain how quantitative variables were handled in the analyses. If applicable, describe which groupings were chosen and why	Methods 'exposures', 'outcomes' and 'covariables', ST4
Statistical methods	12	(*a*) Describe all statistical methods, including those used to control for confounding	Methods 'statistical analyses' & Results
		(*b*) Describe any methods used to examine subgroups and interactions	Methods 'statistical analyses' & 'sensitivity analyses' , Results
		(*c*) Explain how missing data were addressed	Methods 'statistical analyses'
		(*d*) If applicable, explain how loss to follow-up was addressed	N/A
		(*e*) Describe any sensitivity analyses	Methods par 'sensitivity analyses', Results 'robustness of associations with HRQOL'

**Results**			
Participants	13*	(a) Report numbers of individuals at each stage of study—eg numbers potentially eligible, examined for eligibility, confirmed eligible, included in the study, completing follow-up, and analysed	SF1
		(b) Give reasons for non-participation at each stage	SF1
		(c) Consider use of a flow diagram	SF1
Descriptive data	14*	(a) Give characteristics of study participants (eg demographic, clinical, social) and information on exposures and potential confounders	Results par 1 and Table 1
		(b) Indicate number of participants with missing data for each variable of interest	SF7 and SF8
		(c) Summarise follow-up time (eg, average and total amount)	N/A
Outcome data	15*	Report numbers of outcome events or summary measures over time	Results par 1-4, SF1
Main results	16	(*a*) Give unadjusted estimates and, if applicable, confounder-adjusted estimates and their precision (eg, 95% confidence interval). Make clear which confounders were adjusted for and why they were included	Throughout methods and results, Figures and Tables
		(*b*) Report category boundaries when continuous variables were categorized	Methods 'statistical analyses', Table 1, ST4
		(*c*) If relevant, consider translating estimates of relative risk into absolute risk for a meaningful time period	N/A
Other analyses	17	Report other analyses done—eg analyses of subgroups and interactions, and sensitivity analyses	Methods 'statistical analyses' & 'sensitivity analyses', Results par 2-5
**Discussion**			
Key results	18	Summarise key results with reference to study objectives	Discussion par 1
Limitations	19	Discuss limitations of the study, taking into account sources of potential bias or imprecision. Discuss both direction and magnitude of any potential bias	Discussion par 7
Interpretation	20	Give a cautious overall interpretation of results considering objectives, limitations, multiplicity of analyses, results from similar studies, and other relevant evidence	Discussion par 2-6
Generalisability	21	Discuss the generalisability (external validity) of the study results	Discussion par 7
Other information			
Funding	22	Give the source of funding and the role of the funders for the present study and, if applicable, for the original study on which the present article is based	Funding

*: Give information separately for exposed and unexposed groups. Abbreviations: par, paragraph; SF, Supplemental Figure; ST, Supplemental Table.

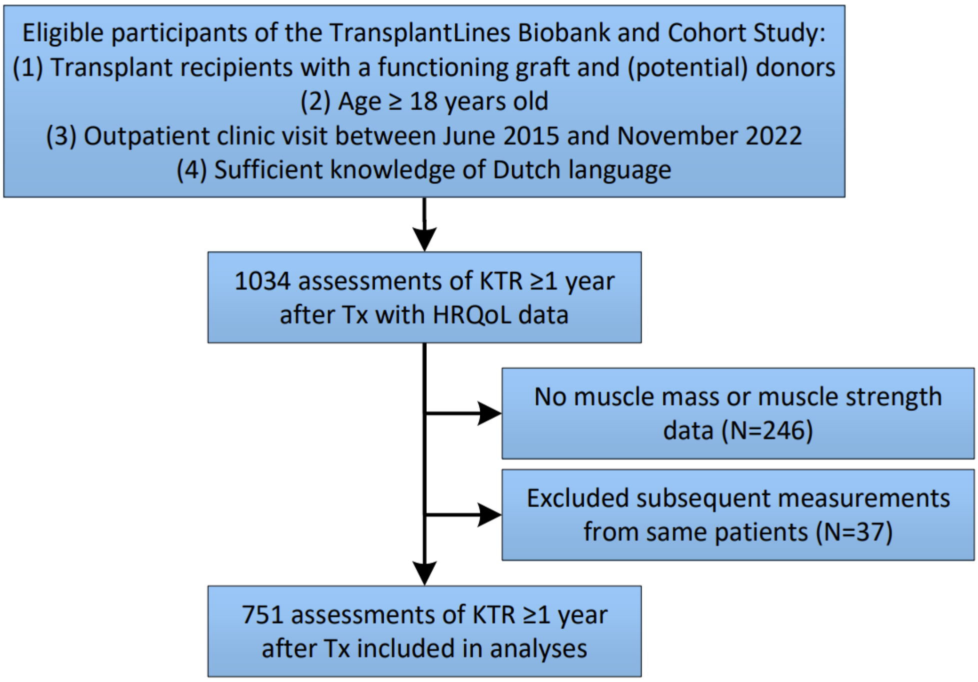

Supplementary Figure S1. CONSORT 2010 flow Diagram. Abbreviations: HRQoL, health-related quality of life; KTR, kidney transplant recipients; Tx, transplantation.

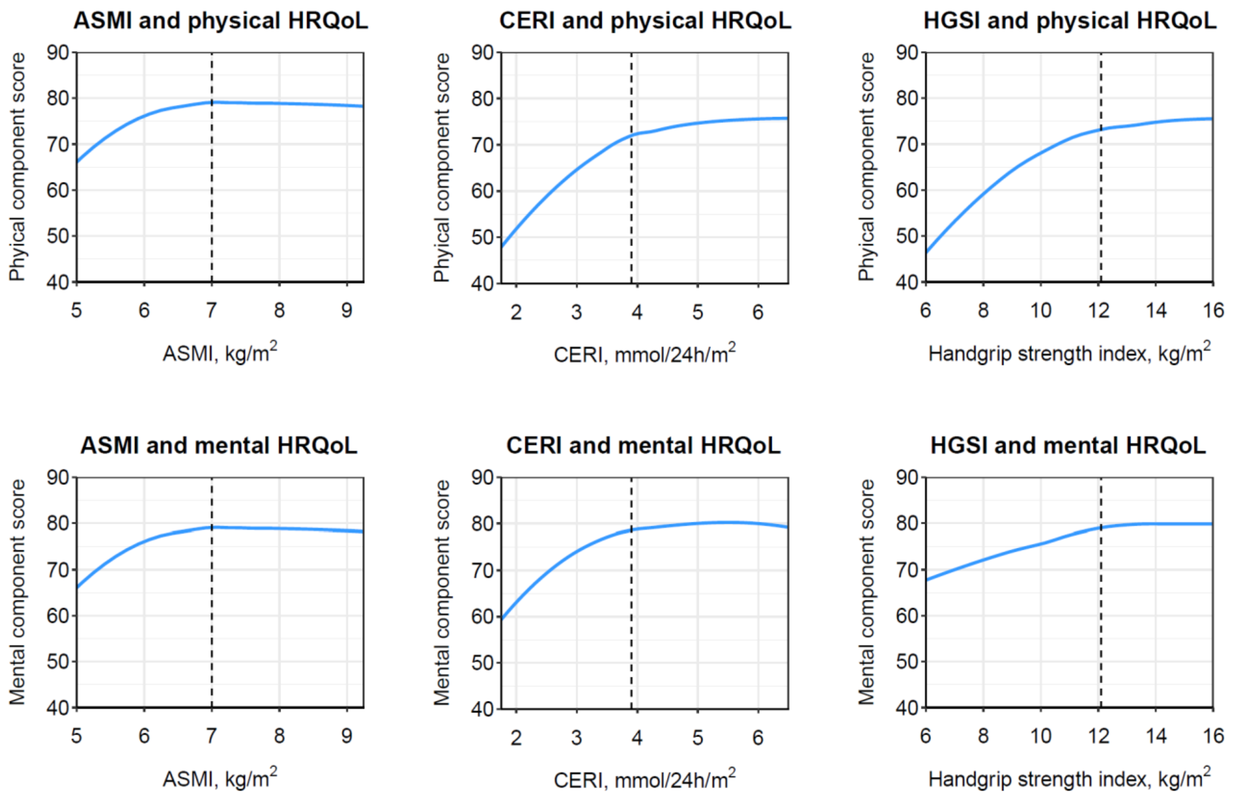

Supplementary Figure S2. Loess curves of the associations between ASMI, CERI or HGSI with physical and mental HRQoL. The dotted line represents the median value of ASMI, CERI and HGSI, which is close to the value before which the associations are strong, and after which the associations are discernible or absent. Abbreviations: Abbreviations: ASMI, appendicular skeletal muscle mass index; CERI, creatinine extraction rate index; HGSI, hand grip strength index; HRQoL, health-related quality of life; KTR, kidney transplant recipients.

Supplementary Table S2. Model fit parameters according to the number of knots in the non-linear models.

	BIC	AIC	P-value 1 vs 2 knots	P-value 2 vs 3 knots	P-value 3 vs 4 knots
Model 5 (of ASMI and HGSI with physical component score)					
With 1 knot (2 degrees of freedom)	6648.46	6532.93			
With 2 knots (3 degrees of freedom)	6656.02	6531.24	0.07		
With 3 knots (4 degrees of freedom)	6663.46	6529.43		0.06	
With 4 knots (5 degrees of freedom)	6674.40	6531.14			0.3
Model 5 (of CERI and HGSI with physical component score)					
With 1 knot (2 degrees of freedom)	6647.32	6531.79			
With 2 knots (3 degrees of freedom)	6653.09	6528.32	0.03		
With 3 knots (4 degrees of freedom)	6665.57	6531.55		0.7	
With 4 knots (5 degrees of freedom)	6676.96	6533.69			0.4
Model 5 (with ASMI and HGSI with mental component score)					
With 1 knot (2 degrees of freedom)	6440.74	6325.21			
With 2 knots (3 degrees of freedom)	6453.51	6328.73	0.8		
With 3 knots (4 degrees of freedom)	6465.02	6331.00		0.4	
With 4 knots (5 degrees of freedom)					0.6
Model 5 (of CERI and HGSI with mental component score)					
With 1 knot (2 degrees of freedom)	6440.35	6324.82			
With 2 knots (3 degrees of freedom)	6452.17	6327.39	0.5		
With 3 knots (4 degrees of freedom)	6465.16	6331.13		0.9	
With 4 knots (5 degrees of freedom)	6478.316	6335.052			1.0

Analysis of Variance (ANOVA) was used to compare linear regression models with natural splines with varying knots, assessing whether the addition of more flexible spline terms significantly improved model fit. A significant p-value (< 0.05) would indicate that the more complex model provides a significantly better fit. Abbreviations: ASMI, appendicular skeletal muscle mass index; CERI, 24-hour urinary creatinine excretion rate index; HGSI, hand grip strength index, HRQOL, health-related quality of life; Std. β, standardized beta.

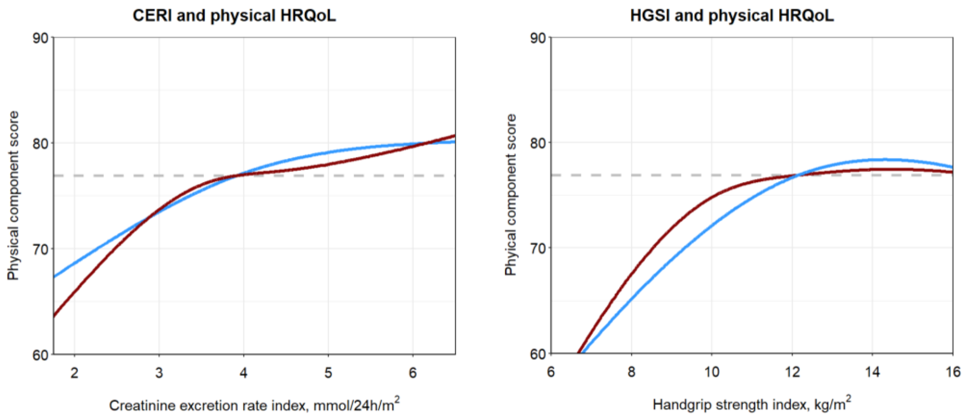

Supplementary Figure S3. Curves of the model including CERI and HGSI with physical HRQoL with 2 (blue) or 3 (red) degrees of freedom. Abbreviations: Abbreviations: CERI, creatinine extraction rate index; HGSI, hand grip strength index; HRQoL, health-related quality of life.

Supplementary Table S3. Characteristics of muscle mass and muscle strength stratified by sex.

	Male KTR N=462	Female KTR N=296	P-value
**Muscle mass and Muscle strength**			
ASM, kg	23.8 ± 3.9	17.7 ± 3.3	<0.001
ASMI, kg/m^2	7.5 ± 1.1	6.4 ± 1.1	<0.001
CER, mmol/24h	13.9 ± 3.7	10.2 ± 2.7	<0.001
CERI, $mmol/24h/m^2$	4.4 ± 1.1	3.7 ± 0.9	<0.001
HGS, kg	44.2 ± 10.3	28.4 ± 7.9	<0.001
HGSI, kg/m^2	13.8 ± 3.0	10.3 ± 2.7	<0.001

Differences were assessed using independent sample T tests. Abbreviations: ASM(I), appendicular skeletal muscle mass (index) CER(I), creatinine excretion rate (index); HGS(I), hand grip strength (index).

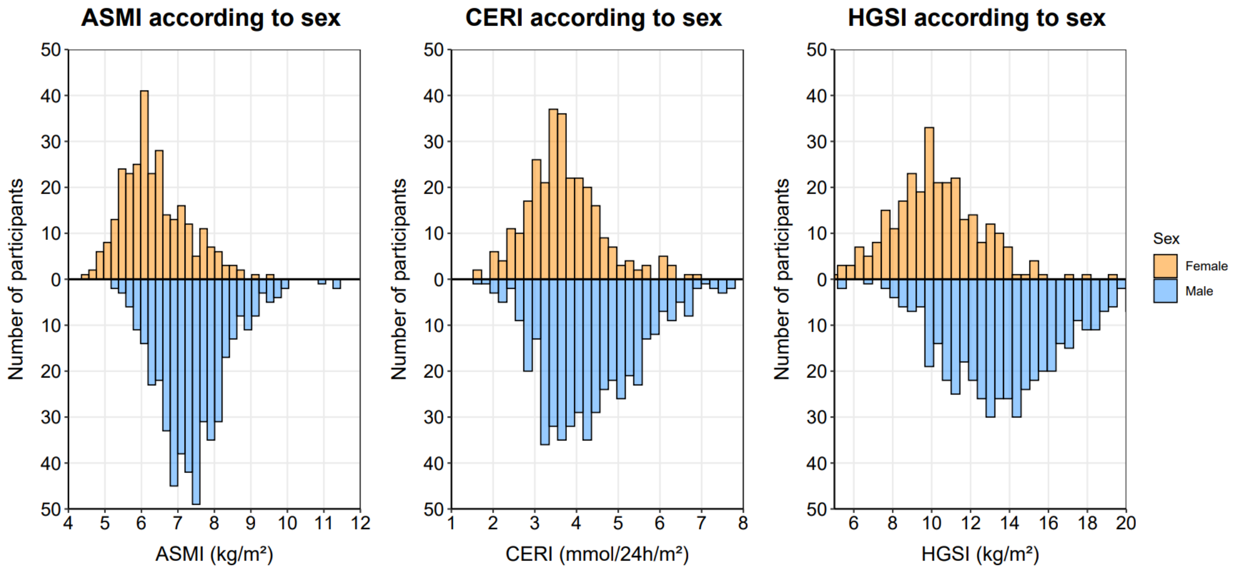

Supplementary Figure S4. Data distribution of ASMI, CERI and HGSI stratified by sex. Abbreviations: ASMI, appendicular skeletal muscle mass index; CERI, creatinine excretion rate index; HGSI, hand grip strength index.

Supplementary Table S4. Characteristics of KTR excluded from and included in analyses.

	KTR excluded from analyses N=283	KTR included in analyses N=751	P-value
**Demographics**			
Female sex, *n* (%)	109 (39)	289 (39)	1.0
Age, years	57 ± 13	56 ± 13	0.3
Weight, kg	84 ± 17	82 ± 16	0.3
Height, cm	175 ± 11	174 ± 9	0.1
Waist circumference, cm	100 ± 14	99 ± 14	0.3
Body surface area, m^2	2.0 ± 0.2	2.0 ± 0.2	0.2
Body mass index, kg/m^2	27 ± 5	27 ± 5	0.9
Diabetes, *n* (%)	74 (26)	210 (28)	0.6
Time since transplantation, *n* (%)			<0.001
≤2 years	144 (51)	321 (43)	
2 to 5 years	56 (20)	108 (14)	
≥5 years	83 (29)	322 (43)	
History of dialysis, *n* (%)	158 (57)	509 (68)	0.001
Living donor, *n* (%)	171 (65)	416 (55)	0.010
**Lifestyle factors**			
Alcohol use, *n* (%)			0.1
No	120 (45)	286 (38)	
<7 units/week	97 (36)	309 (41)	
≥7 units/week	50 (19)	156 (21)	
Smoking status, *n* (%)			0.9
Low	125 (47)	351 (47)	
Medium	108 (40)	306 (41)	
High	36 (13)	94 (13)	
Protein intake, g/day	85 ± 24	84 ± 22	0.9
**Laboratory measurements**			
Hemoglobin, mmol/L	8.2 ± 1.1	8.4 ± 1.1	0.1
Leukocyte count, 10^9/L	7.4 ± 2.4	7.6 ± 2.2	0.3
C-reactive protein, mg/L[†]	2.0 [0.9, 4.9]	1.8 [0.7, 4.4]	0.3
Creatinine, µmol/L	122 [100, 151]	123 [103, 152]	0.4
eGFR, mL/min/1.73m^2	55 ± 19	55 ± 18	0.5
Albumin, g/L	43 ± 3	44 ± 4	0.001
Total protein excretion in 24-hours, g	0.17 [0.12, 0.29]	0.17 [0.12, 0.28]	0.9
**Medication use**			
Prednisolone, *n* (%)	274 (97)	733 (98)	0.6
Calcineurin inhibitor, *n* (%)	251 (89)	618 (82)	0.016
Proliferation inhibitor, *n* (%)	247 (87)	643 (86)	0.6
mTOR inhibitor, *n* (%)	12 (4)	28 (4)	0.8

Normally distributed data were presented as mean ± standard deviation, non-normally distributed data as median [interquartile range] and categorical data were presented as number (valid %). Differences between groups were assessed using independent sample T-tests, Mann-Whitney U tests or chi-square test depending on data-distribution. Data regarding waist circumference, protein intake and total protein excretion in 24-hours were missing in 88 (9%), 101 (10%) and 86 (8%) of the participants, respectively. Other variables were complete or <2% missing was missing. Abbreviations: eGFR, estimated glomerular filtration rate; KTR, kidney transplant recipients; mTOR, mammalian target of rapamycin.

Supplementary Table S5. Statistical significance of potential effect modifications.

		ASMI (kg/m²)	CERI (mmol/24h/m²)	HGSI (kg/m²)
		P-value	P-value	P-value
**Dependent variable: physical component score** *Higher score reflects better physical health-related quality of life*				
Age*variable of interest	St. β_1	0.3	0.5	0.8
	St. β_2	0.4	0.4	0.2
Sex*variable of interest	St. β_1	0.7	0.065	0.9
	St. β_2	0.9	0.2	0.7
eGFR*variable of interest	St. β_1	0.4	0.3	0.8
	St. β_2	0.2	0.076	0.6
Urinary protein excretion*variable of interest	St. β_1	1.0	0.8	1.0
	St. β_2	0.8	0.3	0.5
**Dependent variable: mental component score** *Higher score reflects better mental health-related quality of life*				
Age*variable of interest	St. β_1	0.3	0.4	0.4
	St. β_2	0.7	1.0	0.6
Sex*variable of interest	St. β_1	0.4	0.1	0.8
	St. β_2	0.2	0.4	0.6
eGFR*variable of interest	St. β_1	0.055	0.9	0.059
	St. β_2	0.1	0.3	0.6
Urinary protein excretion*variable of interest	St. β_1	0.7	0.6	0.9
	St. β_2	0.9	0.2	0.9

Abbreviations: ASMI, appendicular skeletal muscle mass index; CERI, creatinine excretion rate index; HGSI, hand grip strength index.

Supplementary Table S6. Ordinary least squares regression analyses on the association of ASMI, CERI and HGSI with HRQoL.

model		ASMI (kg/m²)		CERI (mmol/24h/m²)		HGSI (kg/m²)	
		**Std. β [95% CI]**	**P-value**	**Std. β [95% CI]**	**P-value**	**Std. β [95% CI]**	**P-value**
**Physical component score** *Higher score reflects better physical HRQoL*							
**0**	St. β_1	1.06 [0.41, 1.71]	0.001	2.25 [1.55, 2.95]	<0.001	3.55 [2.79, 4.30]	<0.001
	St. β_2	0.07 [-0.16, 0.30]	0.6	0.66 [0.40, 0.91]	<0.001	0.79 [0.55, 1.04]	<0.001
**1**	St. β_1	2.25 [1.43, 3.07]	<0.001	2.18 [1.47, 2.89]	<0.001	3.62 [2.79, 4.44]	<0.001
	St. β_2	0.64 [0.34, 0.93]	<0.001	0.75 [0.47, 1.03]	<0.001	0.85 [0.55, 1.15]	<0.001
**2**	St. β_1	2.02 [1.20, 2.84]	<0.001	2.02 [1.31, 2.73]	<0.001	3.41 [2.59, 4.24]	<0.001
	St. β_2	0.56 [0.26, 0.85]	<0.001	0.70 [0.42, 0.97]	<0.001	0.76 [0.46, 1.06]	<0.001
**3**	St. β_1	1.82 [1.01, 2.64]	<0.001	1.67 [0.96, 2.39]	<0.001	3.17 [2.33, 4.00]	<0.001
	St. β_2	0.49 [0.19, 0.78]	0.001	0.58 [0.30, 0.86]	<0.001	0.62 [0.32, 0.92]	<0.001
**4**	St. β_1	1.58 [0.75, 2.41]	<0.001	1.50 [0.72, 2.29]	<0.001	3.07 [2.24, 3.90]	<0.001
	St. β_2	0.39 [0.10, 0.69]	0.010	0.48 [0.14, 0.82]	0.006	0.57 [0.26, 0.87]	<0.001
**5a**	St. β_1	1.00 [0.17, 1.84]	0.023	1.02 [0.24, 1.81]	0.010	-	-
	St. β_2	0.22 [-0.09, 0.51]	0.2	0.38 [0.04, 0.72]	0.029	-	-
**5b**	St. β_1	-	-	-	-	2.83 [1.98, 3.69]	<0.001
	St. β_2	-	-	-	-	0.48 [0.17, 0.79]	0.002
**5c**	St. β_1	-	-	-	-	2.84 [2.00, 3.69]	<0.001
	St. β_2	-	-	-	-	0.46 [0.15, 0.78]	0.004
**Mental component score** *Higher score reflects better mental HRQoL*							
**0**	St. β_1	1.54 [0.89, 2.19]	<0.001	1.98 [1.28, 2.68]	<0.001	2.00 [1.21, 2.79]	<0.001
	St. β_2	0.22 [-0.01, 0.45]	0.057	0.42 [0.16, 0.68]	0.002	0.45 [0.19, 0.71]	<0.001
**1**	St. β_1	2.22 [1.37, 3.08]	<0.001	2.24 [1.51, 2.97]	<0.001	2.01 [1.12, 2.90]	<0.001
	St. β_2	0.53 [0.22, 0.84]	<0.001	0.61 [0.31, 0.89]	<0.001	0.52 [0.20, 0.84]	0.001
**2**	St. β_1	2.10 [1.24, 2.96]	<0.001	2.16 [1.42, 2.89]	<0.001	1.88 [0.99, 2.77]	<0.001
	St. β_2	0.49 [0.18, 0.80]	0.002	0.58 [0.29, 0.87]	<0.001	0.47 [0.15, 0.79]	0.004
**3**	St. β_1	1.96 [1.10, 2.83]	<0.001	1.92 [1.17, 2.68]	<0.001	1.72 [0.81, 2.62]	<0.001
	St. β_2	0.45 [0.14, 0.77]	0.004	0.53 [0.23, 0.82]	<0.001	0.38 [0.05, 0.71]	0.022
**4**	St. β_1	1.96 [1.10, 2.83]	<0.001	1.80 [0.97, 2.62]	<0.001	1.61 [0.71, 2.52]	<0.001
	St. β_2	0.45 [0.14, 0.77]	0.004	0.45 [0.09, 0.81]	0.014	0.32 [-0.00, 0.66]	0.050
**5a**	St. β_1	1.79 [0.92, 2.66]	<0.001	1.57 [0.73, 2.42]	<0.001	-	-
	St. β_2	0.38 [0.07, 0.71]	0.017	0.40 [0.04, 0.77]	0.033	-	-
**5b**	St. β_1	-	-	-	-	1.28 [0.36, 2.20]	0.006
	St. β_2	-	-	-	-	0.22 [-0.12, 0.56]	0.2
**5c**	St. β_1	-	-	-	-	1.27 [0.36, 2.19]	0.006
	St. β_2	-	-	-	-	0.21 [-0.13, 0.55]	0.2

Model 1: Adjusted for age, sex, estimated glomerular filtration rate, total protein excretion in 24-hours, and waist circumference. **Model 2**: As model 1, additionally adjusted for history of dialysis, living donor, and time since transplantation. **Model 3**: As model 2, additionally adjusted for alcohol use, smoking status, diabetes, C-reactive protein and hemoglobin. **Model 4**: As model 3, additionally adjusted for protein intake. **Model 5**: As model 4, additionally adjusted for HGSI (a), ASMI (b) or CERI (c). Because of collinearity between ASMI and CERI, both variables were not adjusted in one model, but were adjusted separately. **Abbreviations**: ASMI, appendicular skeletal muscle mass index; CERI, 24-hour urinary creatinine excretion rate index; HGSI, hand grip strength index, HRQoL, health-related quality of life; Std. β, standardized beta.

Supplementary Table S7. Associations of ASMI, CERI or HSGI with FEV_1, and their relationship with HRQoL in mutually adjusted analyses.

**Dependent variable: FEV_1**						
Higher value indicates better FEV_1						
Variable of interest:	ASMI (kg/m²)		CERI (mmol/24h/m²)		HGSI (kg/m²)	
	Std. β [95% CI]	P-value	Std. β [95% CI]	P-value	Std. β [95% CI]	P-value
Base model + variable of interest						
St. β variable of interest	0.11 [0.04, 0.18]	0.002	0.07 [-0.01, 0.14]	0.071	0.14 [0.07, 0.20]	<0.001
**Dependent variable: physical component score**						
Higher score reflects better physical health-related quality of life						
	Std. β [95% CI]	P-value				
Base model + FEV_1						
St. β FEV_1	0.12 [0.01, 0.23]	0.030				
Variable of interest:	ASMI (kg/m²)		CERI (mmol/24h/m²)		HGSI (kg/m²)	
	Std. β [95% CI]	P-value	Std. β [95% CI]	P-value	Std. β [95% CI]	P-value
Base model + variable of interest						
St. β_1 variable of interest	1.78 [0.93, 2.62]	<0.001	1.88 [0.99, 2.77]	<0.001	3.11 [2.28, 3.94]	<0.001
St. β_2 variable of interest	0.47 [0.16, 0.77]	0.003	0.48 [0.15, 0.81]	0.005	0.57 [0.27, 0.88]	<0.001
Base model + FEV_1 and variable of interest						
St. β FEV_1	0.09 [-0.02, 0.20]	0.094	0.10 [-0.01, 0.21]	0.065	0.09 [-0.01, 0.20]	0.092
St. β_1 variable of interest	1.84 [0.93, 2.76]	<0.001	1.76 [0.90, 2.61]	<0.001	2.92 [2.05, 3.80]	<0.001
St. β_2 variable of interest	0.45 [0.12, 0.78]	0.007	0.65[0.27, 1.03]	<0.001	0.52 [0.19, 0.85]	0.002
**Dependent variable: mental component score**						
Higher score reflects better mental health-related quality of life						
	Std. β [95% CI]	P-value				
Base model + FEV_1						
St. β FEV_1	0.11 [-0.00, 0.22]	0.060				
Variable of interest:	ASMI (kg/m²)		CERI (mmol/24h/m²)		HGSI (kg/m²)	
	Std. β [95% CI]	P-value	Std. β [95% CI]	P-value	Std. β [95% CI]	P-value
Base model + variable of interest						
St. β_1 variable of interest	1.80 [0.91, 0.70]	<0.001	1.91 [0.96, 2.85]	<0.001	1.64 [0.74, 2.55]	<0.001
St. β_2 variable of interest	0.39 [0.07, 0.71]	0.018	0.39 [0.03, 0.74]	0.031	0.33 [-0.01, 0.66]	0.054
Base model + FEV_1 and variable of interest						
St. β FEV_1	0.09 [-0.03, 0.20]	0.1	0.09 [-0.02, 0.21]	0.1	0.09 [-0.02, 0.21]	0.1
St. β_1 variable of interest	1.76 [0.82, 2.70]	<0.001	1.66 [0.77, 2.56]	<0.001	1.44 [0.51, 2.37]	0.002
St. β_2 variable of interest	0.26 [-0.08, 0.60]	0.1	0.48 [0.08, 0.88]	0.018	0.34 [-0.02, 0.69]	0.064

Variables included in the base model are age, sex, estimated glomerular filtration rate and total protein excretion in 24-hours, waist circumference, history of dialysis, living donor, time since transplantation, alcohol use, smoking status, diabetes, C-reactive protein, hemoglobin, protein intake and height. Analyses with FEV_1 included in the model were performed in 667 (89%) participants, due to missing data regarding FEV_1. Abbreviations: CERI, 24-hour urinary creatinine excretion rate index; FEV_1, forced expiratory volume in 1 second; HGSI, hand grip strength index; HRQoL, health-related quality of life.

Supplementary Table S8. Associations of ASMI, CERI or HSGI with airflow limitation and their relationship with HRQoL in mutually adjusted analyses.

**Dependent variable: airflow limitation**						
Variable of interest:	ASMI (kg/m²)		CERI (mmol/24h/m²)		HGSI (kg/m²)	
	OR [95% CI]	P-value	OR [95% CI]	P-value	OR [95% CI]	P-value
Base model + variable of interest						
St. β variable of interest	0.66 [0.47, 0.88]	0.009	0.81 [0.61, 1.06]	0.1	0.73 [0.56, 0.93]	0.014
**Dependent variable: physical component score**						
Higher score reflects better physical HRQoL						
	Std. β [95% CI]	P-value				
Base model + airflow limitation						
St. β airflow limitation	-0.15 [-0.32, 0.02]	0.086				
Variable of interest:	ASMI (kg/m²)		CERI (mmol/24h/m²)		HGSI (kg/m²)	
	Std. β [95% CI]	P-value	Std. β [95% CI]	P-value	Std. β [95% CI]	P-value
Base model + variable of interest						
St. β_1 variable of interest	1.58 [0.75, 2.41]	<0.001	1.50 [0.72, 2.29]	<0.001	3.07 [2.24, 3.90]	<0.001
St. β_2 variable of interest	0.39 [0.10, 0.69]	0.010	0.48 [0.14, 0.82]	0.006	0.57 [0.26, 0.87]	<0.001
Base model + airflow limitation and variable of interest						
St. β airflow limitation	-0.12 [-0.29, 0.05]	0.2	-0.13 [-0.30, 0.04]	0.1	-0.14 [-0.30, 0.03]	0.1
St. β_1 variable of interest	1.74 [0.86, 2.62]	<0.001	1.60 [0.77, 2.44]	0.003	2.97 [2.10, 3.84]	<0.001
St. β_2 variable of interest	0.42 [0.10, 0.74]	0.011	0.55 [0.18, 0.91]	0.1	0.53 [0.20, 0.86]	0.002
**Dependent variable: mental component score**						
Higher score reflects better mental HRQoL						
	Std. β [95% CI]	P-value				
Base model + airflow limitation						
St. β airflow limitation	-0.14 [-0.31, 0.03]	0.1				
Variable of interest:	ASMI (kg/m²)		CERI (mmol/24h/m²)		HGSI (kg/m²)	
	Std. β [95% CI]	P-value	Std. β [95% CI]	P-value	Std. β [95% CI]	P-value
Base model + variable of interest						
St. β_1 variable of interest	1.96 [1.10, 2.83]	<0.001	1.80 [0.97, 2.62]	<0.001	1.61 [0.71, 2.52]	<0.001
St. β_2 variable of interest	0.45 [0.14, 0.77]	0.004	0.45 [0.09, 0.81]	0.014	0.32 [-0.00, 0.66]	0.050
Base model + airflow limitation and variable of interest						
St. β airflow limitation	-0.13 [-0.30, 0.05]	0.2	-0.13 [-0.30, 0.05]	0.2	-0.13 [-0.31, 0.04]	0.1
St. β_1 variable of interest	1.78 [0.86, 2.69]	<0.001	1.65 [0.78, 2.51]	<0.001	1.48 [0.56, 2.41]	0.002
St. β_2 variable of interest	0.26 [-0.07, 0.60]	0.1	0.46 [0.08, 0.84]	0.017	0.36 [0.01, 0.71]	0.045

Variables included in the base model are age, sex, estimated glomerular filtration rate and total protein excretion in 24-hours, waist circumference, history of dialysis, living donor, time since transplantation, alcohol use, smoking status, diabetes, C-reactive protein, hemoglobin, protein intake and height. Analyses with airflow limitation included in the model were performed in 667 (89%) participants, due to missing data regarding forced expiratory volume in 1 second. Airflow limitation was defined as a forced expiratory volume in 1 second <5th percentile of an age-, sex-, height- and ethnicity-matched reference population. Abbreviations: CERI, 24-hour urinary creatinine excretion rate index; HGSI, hand grip strength index; HRQoL, health-related quality of life.

Supplementary Table S9. Associations of ASMI, CERI or HSGI with fatigue severity and their relationship with HRQoL in mutually adjusted analyses.

**Dependent variable: fatigue severity**						
Higher score indicates more fatigue						
Variable of interest:	ASMI (kg/m²)		CERI (mmol/24h/m²)		HGSI (kg/m²)	
	OR [95% CI]	P-value	OR [95% CI]	P-value	OR [95% CI]	P-value
Base model + variable of interest						
St. β variable of interest	-0.06 [-0.15, 0.03]	0.2	-0.09 [-0.09, 0.01]	0.074	-0.12 [-0.21, -0.03]	0.011
**Dependent variable: physical component score**						
Higher score reflects better physical HRQoL						
	Std. β [95% CI]	P-value				
Base model + fatigue severity						
St. β fatigue severity	-0.65 [-0.70, -0.60]	<0.001				
Variable of interest:	ASMI (kg/m²)		CERI (mmol/24h/m²)		HGSI (kg/m²)	
	Std. β [95% CI]	P-value	Std. β [95% CI]	P-value	Std. β [95% CI]	P-value
Base model + variable of interest						
St. β_1 variable of interest	1.58 [0.75, 2.41]	<0.001	1.50 [0.72, 2.29]	<0.001	3.07 [2.24, 3.90]	<0.001
St. β_2 variable of interest	0.39 [0.10, 0.69]	0.010	0.48 [0.14, 0.82]	0.006	0.57 [0.26, 0.87]	<0.001
Base model + airflow limitation and variable of interest						
St. β fatigue severity	-0.64 [-0.70, -0.59]	<0.001	-0.64 [0.70, -0.59]	<0.001	-0.63 [-0.68, -0.58]	<0.001
St. β_1 variable of interest	0.78 [0.30, 1.56]	0.014	0.78 [0.34, 1.55]	0.010	2.03 [1.40, 2.66]	<0.001
St. β_2 variable of interest	0.21 [0.04, 0.49]	0.062	0.33 [0.17, 0.70]	0.012	0.32 [0.09, 0.55]	0.006
**Dependent variable: mental component score**						
Higher score reflects better mental HRQoL						
	Std. β [95% CI]	P-value				
Base model + fatigue severity						
St. β fatigue severity	-0.67 [-0.73, -0.61]	<0.001				
Variable of interest:	ASMI (kg/m²)		CERI (mmol/24h/m²)		HGSI (kg/m²)	
	Std. β [95% CI]	P-value	Std. β [95% CI]	P-value	Std. β [95% CI]	P-value
Base model + variable of interest						
St. β_1 variable of interest	1.96 [1.10, 2.83]	<0.001	1.80 [0.97, 2.62]	<0.001	1.61 [0.71, 2.52]	<0.001
St. β_2 variable of interest	0.45 [0.14, 0.77]	0.004	0.45 [0.09, 0.81]	0.014	0.32 [-0.00, 0.66]	0.050
Base model + airflow limitation and variable of interest						
St. β fatigue severity	-0.66 [-0.72, -0.60]	<0.001	-0.66 [-0.72, -0.60]	<0.001	-0.66 [-0.72, -0.61]	<0.001
St. β_1 variable of interest	0.88 [0.21, 1.55]	0.011	0.95 [0.31, 1.59]	0.004	0.50 [-0.20, 1.21]	0.2
St. β_2 variable of interest	0.16 [-0.72, -0.60]	0.2	0.26 [-0.01, 0.54]	0.062	0.07[-0.18, 0.33]	0.6

Variables included in the base model are age, sex, estimated glomerular filtration rate and total protein excretion in 24-hours, waist circumference, history of dialysis, living donor, time since transplantation, alcohol use, smoking status, diabetes, C-reactive protein, hemoglobin, protein intake and height. Abbreviations: CERI, 24-hour urinary creatinine excretion rate index; HGSI, hand grip strength index; HRQoL, health-related quality of life.

Supplementary Table S10. Analyses to assess the robustness of ordinary least squares regression analyses on the association of muscle mass and muscle strength with HRQoL.

Model 5		**ASM(I)** *Adjusted for HGSI*		**CER(I)** *Adjusted for HGSI*		**HGS(I)** *Adjusted for ASM(I)*		**HGS(I)** *Adjusted for CER(I)*	
		**Std. β [95% CI]**	**P-value**	**Std. β [95% CI]**	**P-value**	**Std. β [95% CI]**	**P-value**	**Std. β [95% CI]**	**P-value**
**Physical component score** *Higher score reflects better physical HRQoL*									
Primary analyses	St. β_1	1.00 [0.17, 1.84]	0.023	1.02 [0.24, 1.81]	0.010	2.83 [1.98, 3.69]	<0.001	2.84 [2.00, 3.69]	<0.001
	St. β_2	0.22 [-0.09, 0.51]	0.2	0.38 [0.04, 0.72]	0.029	0.48 [0.17, 0.79]	0.002	0.46 [0.15, 0.78]	0.004
Unindexed analyses[1]	St. β_1	0.95 [-0.12, 2.02]	0.082	1.25 [0.39, 2.11]	0.005	2.48 [1.61, 3.34]	<0.001	2.43 [1.60, 3.26]	<0.001
	St. β_2	0.18 [-0.20, 0.56]	0.4	0.47 [0.07, 0.86]	0.020	0.49 [0.16, 0.83]	0.004	0.43 [0.10, 0.76]	0.011
Without knots boundaries	St. β_1	0.79 [-0.21, 1.79]	0.1	1.03 [0.24, 1.83]	0.011	2.40 [1.57, 3.22]	<0.001	2.39 [1.56, 3.21]	<0.001
	St. β_2	-0.97 [-2.20, 0.26]	0.1	0.28 [-0.37, 0.93]	0.4	-0.89 [-1.68, -0.09]	0.029	-0.89 [-1.68, -0.09]	0.089
After exclusion of KTR with	St. β_1	1.15 [0.26, 2.03]	0.011	1.20 [0.37, 2.03]	0.005	3.02 [1.95, 4.08]	<0.001	3.10 [2.05, 4.15]	<0.001
eGFR ≤30 mL/min/1.73m^2[2]	St. β_2	0.25 [-0.16, 0.57]	0.1	0.40 [0.04, 0.76]	0.028	0.56 [0.18, 0.94]	0.004	0.55 [0.18, 0.92]	0.004
After exclusion of KTR with an	St. β_1	1.05 [0.21, 1.89]	0.015	1.21 [0.42, 2.01]	0.002	2.88 [1.89, 3.88]	<0.001	2.93 [1.94, 3.92]	<0.001
age ≥75 years[3]	St. β_2	0.21 [0.09, 0.52]	0.2	0.44 [0.10, 0.77]	0.011	0.55 [0.20, 0.91]	0.002	0.52 [0.17, 0.87]	0.004
**Mental component score** *Higher score reflects better mental HRQoL*									
Primary analyses	St. β_1	1.79 [0.92, 2.66]	<0.001	1.57 [0.73, 2.42]	<0.001	1.28 [0.36, 2.20]	0.006	1.27 [0.36, 2.19]	0.006
	St. β_2	0.38 [0.07, 0.71]	0.017	0.40 [0.04, 0.77]	0.033	0.22 [-0.12, 0.56]	0.2	0.21 [-0.13, 0.55]	0.2
Unindexed analyses[1]	St. β_1	1.02 [-0.14, 2.9]	0.087	1.64 [0.72, 2.57]	<0.001	1.01 [0.08, 1.94]	0.034	0.87 [-0.02, 1.77]	0.056
	St. β_2	0.17 [-0.25, -0.58]	0.43	0.43 [0.00, 0.86]	0.048	0.24 [-0.13, 0.60]	0.2	0.17 [-0.18, 0.53]	0.3
Without knots boundaries	St. β_1	1.06 [-0.03, 2.15]	0.056	1.52 [0.66, 2.38]	<0.001	1.12 [0.23, 2.02]	0.014	1.09 [0.20, 1.98]	0.016
	St. β_2	-1.57 [-2.90, -0.24]	0.021	0.01 [-0.70, 0.71]	1.0	-0.48 [-1.34, 0.39]	0.3	-0.39 [-1.27, 0.47]	0.4
After exclusion of KTR with	St. β_1	1.67 [0.71, 2.62]	<0.001	1.55 [0.66, 2.45]	<0.001	1.51 [0.37, 2.66]	0.010	1.63 [0.50, 2.77]	0.005
eGFR ≤30 mL/min/1.73m^2[2]	St. β_2	0.36 [-0.02, 0.69]	0.039	0.37 [-0.02, 0.75]	0.063	0.27 [-0.13, 0.67]	0.2	0.28 [-0.12, 0.68]	0.2
After exclusion of KTR with an	St. β_1	1.54 [0.63, 2.46]	<0.001	1.53 [0.66, 2.40]	<0.001	1.05 [0.04, 2.14]	0.058	1.13 [0.05, 2.21]	0.041
age ≥75 years[3]	St. β_2	0.28 [-0.06, 0.61]	0.1	0.40 [0.03, 0.77]	0.033	0.22 [-0.17, 0.61]	0.27	0.20 [-0.19, 0.59]	0.3

Variables included in model 4 are the variable of interest, age, sex, estimated glomerular filtration rate and total protein excretion in 24-hours, waist circumference, history of dialysis, living donor, time since transplantation, alcohol use, smoking status, diabetes, C-reactive protein, hemoglobin, and protein intake. [1]: Unindexed analyses refers to the measurements not being indexed to height squared, thus using CER and HGS. [2]: N=679. [3]: N=716. Abbreviations: CERI, 24-hour urinary creatinine excretion rate index; HGSI, hand grip strength index, HRQOL: health-related quality of life, KTR: kidney transplant recipients.
